# Acceptance and commitment therapy group protocol for caregivers of anxious youth: an open trial pilot study

**DOI:** 10.3389/frcha.2024.1347295

**Published:** 2024-09-27

**Authors:** Jacquelyn N. Raftery-Helmer, Ashley S. Hart, Madeline R. Levitt, Steven M. Hodge, Lisa W. Coyne, Phoebe S. Moore

**Affiliations:** ^1^ Department of Psychology, Worcester State University, Worcester, MA, United States; ^2^ Department of Psychiatry, University of Massachusetts Chan Medical School, Worcester, MA, United States; ^3^ Department of Psychiatry, Weill Cornell Medical College, New York, NY, United States; ^4^ Department of Psychiatry, Harvard Medical School, Boston, MA, United States

**Keywords:** child anxiety, acceptance and commitment therapy, parenting, child internalizing problems, cognitive fusion

## Abstract

**Introduction:**

Anxiety disorders are common, distressing, and impairing for children and families. Cognitive-behavioral interventions targeting the role of family interactions in child anxiety treatment may be limited by lack of attention to antecedents to parental control; specifically, internal parent factors such as experiential avoidance and cognitive fusion. This pilot study evaluates the preliminary efficacy of a group-delivered caregiver treatment program, ACT for Parents of Anxious Children (ACT-PAC) that targets parental experiential avoidance, cognitive fusion, and child internalizing symptoms.

**Methods:**

Twenty-three youth ages 7–17 years with a primary anxiety disorder diagnosis and their primary caregiver participated in six one-hour, weekly group treatment sessions. Parents and children reported on child symptomatology and parents reported on parent symptomatology and quality of life at two assessment points: within one week before ACT-PAC treatment and within one week after treatment. Parents self-reported on parental internal processes specifically targeted by ACT (e.g., cognitive fusion) weekly during the 6-week treatment.

**Results:**

Results support the feasibility and acceptability of ACT-PAC and indicate reductions in parents' cognitive fusion and child internalizing symptoms.

## 1 Introduction

Anxiety disorders are the most commonly experienced mental health problems for children under 18, affecting up to 20% of youth (1, 2) and representing an early low-degree expression of successive, more severe mental health problems (3–7). While empirically supported treatments for child anxiety exist, up to 40% of children show minimal improvement following treatment (8). Given that treatment-interfering parent-child interactions, specifically parental control or lack of autonomy-granting (9, 10) have been found to characterize anxiety affected families (11), parenting behavior represents a promising target for improving treatment outcomes. However, research aimed at enhancing psychotherapy outcomes by involving parents in treatment has generally been disappointing (12).

Most parent-focused interventions for childhood anxiety have one notable limitation in that they have assumed that parents have the psychological flexibility to make behavior changes, such that they are able to engage in flexible patterns of behavior that support their learning more effective parenting strategies (13). Existing interventions have largely been instructive—coaching parents, for instance, on how to be more involved in treatment, training them to serve as lay CBT therapists, or teaching generic parenting skills [e.g., (14, 15)]. Even the few interventions that have directly targeted parental responses to child anxiety (e.g., The Space Program; (16); Family-Based Cognitive-Behavioral Therapy; (17)) and that have been effective in comparison to active control treatments (18) and non-inferior to child-based Cognitive Behavioral Therapy (19) lack attention to the psychological barriers, such as experiential avoidance [parents attempt to reduce their own emotional reactivity or distress upon seeing their child anxious; (20)] and cognitive fusion [parents responding to their thoughts as literal content; (21)] that might interfere with parents implementing recommended behavioral changes.

Acceptance and Commitment Therapy [ACT; (22)], a treatment model which incorporates mindfulness and acceptance, has substantial promise for enhancing behavior change in parents as a means of improving treatment outcomes for anxious youth (23). ACT is grounded in relational frame theory and as such highlights the role of language and verbal experiences in unhelpful behavioral processes. The efficacy of ACT has been well-documented for a variety of psychological conditions in adults (24–26) and shows promise for alleviating symptoms of mental health problems in youth, including those with anxiety disorders. Swain et al. (27) conducted a randomized controlled trial of ACT and CBT for anxious children; both treatments used a “parent-as coach” approach, teaching parents the same therapeutic skills children were learning. Children in both ACT and CBT groups showed reductions in clinical severity and symptom ratings (28), with gains maintained at three-month follow-up. While promising, the intervention did not directly target or measure parent psychological flexibility as a variable that may impact the ability to respond effectively to one's anxious child. Several ACT protocols have been designed and tested specifically for parents of youth with chronic physical [e.g., cerebral palsy; (29)] or psychological conditions [e.g., autism (30, 31) and aggression^1^] and have shown success in improving parent adjustment, well-being, and psychological flexibility (31) Given the role of parent behavior in pediatric anxiety disorders, and in light of evidence pointing to the utility of parent-focused ACT in improving psychological symptoms in children and psychological well-being and flexibility in parents, the present study piloted a group-delivered ACT for Parents of Anxious Children (ACT-PAC) protocol (see text footnote 1); the protocol is freely available to the Association for Contextual Behavioral Science community) that would serve as an adjunctive treatment to child-alone treatment for anxiety. The primary aim of this study was to evaluate the feasibility and acceptability of ACT-PAC. Our second, exploratory aim, was to assess within-subject change in (1) child symptomatology, (2) parent internal processes specifically targeted by ACT, namely experiential avoidance and cognitive fusion, and (3) parent symptomatology and quality of life.

## 2 Methods and materials

### 2.1 Participants

Twenty three youth ages 7–17 years (14 males, 9 females; mean age 12.7) and their primary caregiver (20 mothers, 3 fathers, mean age 45 years) participated in this study. All participants were European American. Parents were highly educated, with 17% completing some college, 48% completing college, and 26% reporting post college education (education was not reported for 2 participants). All children had a primary anxiety disorder diagnosis as determined by the ADIS-C/P (The Anxiety Disorders Interview Schedule-Child and Parent version), and most common diagnoses were Specific Phobia (96%), Generalized Anxiety Disorder (52%), Obsessive-Compulsive Disorder (48%), Social Phobia (43%), and Separation Anxiety Disorder (22%). At study entry, 73% of children were taking psychiatric medication and 70% of children were engaged in psychotherapy.

### 2.2 Procedure

Thirteen participants were recruited using flyers that targeted parents with a child struggling with anxiety or parents struggling to help their child cope with anxiety. Flyers were posted at a university based Pediatric OCD and Anxiety Disorders Clinic and advertised a 6-week parenting group and child anxiety assessment. Two participants were on the waitlist to begin treatment at the Pediatric OCD and Anxiety Disorders Clinic and were offered the opportunity to participate in this study while they awaited treatment. Eight participants were recruited through community therapist referrals, in which community therapists were asked by the research team to make referrals to parents looking for additional ways to support their child with anxiety. Families who expressed interest completed a brief phone screen about the child's anxiety diagnosis, treatment, and symptoms. Those who reported a previous anxiety disorder diagnosis, current or previous therapy for anxiety-related concerns, and/or high interference caused by anxiety symptoms participated in a diagnostic assessment using the Anxiety Disorders Interview Schedule for DSM-IV: Child and Parent Version (ADIS-C/P). Based on the results of the ADIS-/CP, study clinicians determined whether children met criteria for the study, which required having a primary anxiety disorder diagnosis (diagnosis causing the most functional interference; children could have comorbid diagnoses). The following diagnosis met inclusion criteria: generalized anxiety disorder, obsessive-compulsive disorder, specific phobia, social anxiety disorder, separation anxiety disorder and agoraphobia. Comorbid diagnoses included persistent depressive disorder, major depressive disorder, ADHD inattentive type, ADHD combined type, and oppositional defiant disorder. Children were excluded if they needed inpatient services, were diagnosed with psychosis or if they experienced a new onset of suicidal ideation. Parents were excluded if they experienced a new onset of suicidal ideation. All procedures were approved by the University of Massachusetts Chan Medical School Institutional Review Board.

#### 2.2.1 Assessment schedule

We used a within-subject repeated measures design with two assessment points: within one week before ACT-PAC treatment (pre-treatment) and within one week after treatment (post-treatment). The ACT Parenting Measure and Cognitive Fusion Questionnaire (CFQ) were administered at pre-treatment, post-treatment, and weekly during the 6 week treatment. While parents completed their questionnaires, children who requested help with reading the questionnaires were given assistance by the study coordinator. Participating families were given $20 for each assessment (pre-treatment, post-treatment). See Table 1 for a list of study measures.

**Table 1:** Summary schedule of data collected.

Measure	Pre-treatment	Post-treatment	Weekly	Informant
Demographics form	X			Caregiver
Child behavior checklist	X	X		Caregiver
Screen for child anxiety related emotional disorders	X	X		Caregiver Youth
Depression, anxiety, and stress scale	X	X		Caregiver
Quality of life enjoyment and satisfaction questionnaire-short form	X	X		Caregiver
Multidimensional experiential avoidance questionnaire	X	X		Caregiver
Cognitive fusion questionnaire	X	X	X	Caregiver
ACT parenting measure	X	X	X	Caregiver
Parental acceptance questionnaire	X	X		Caregiver
Client satisfaction questionnaire		X		Caregiver
Qualitative feedback interview		X		Caregiver
Youth self report	X	X		Youth

#### 2.2.2 Intervention

Four groups of 6–7 parents participated in six one-hour, weekly group treatment sessions adapted from a more general ACT protocol, ACT for Parents (see text footnote 1). Groups were created based on date of enrollment, with each group launching after 6–7 participants were enrolled in the study. The ACT for Parents of Anxious Children Protocol (ACT-PAC) targeted psychological processes (e.g., cognitive fusion, experiential avoidance) hypothesized to impede parents from reducing behavior known to contribute to child anxiety (e.g., intrusiveness/restriction of autonomy). Treatment modules focused on mindfulness, cognitive defusion, acceptance, values, committed action, and self-care in the context of parenting a child with an anxiety disorder. (See Table 2 for additional information on intervention components).

**Table 2:** ACT for parents of anxious children intervention components.

Session number and title	Session objectives	Home practice
1. Mindfulness: finding stillness	Listening to parenting obstacles Noticing and normalizing parenting stress Introducing present moment awareness	Mindfulness in daily life
2. Defusion: weathering thoughts & feelings	Listening to difficult parenting thoughts & feelings Introducing how the mind works: Fusion, experiential avoidance & defusion	Defusion/Weathering parent-child interaction
3. The matrix: moving towards vs. moving away	Listening to challenging interactions with your child Introducing concept of workability/acceptance in parent-child interaction	Tracking towards (values-oriented) vs. away (experientially avoidant) behavior
4. Value/committed action: doing what matters	Listening to core parenting values Introducing valuing/committed action in parenting consistency	Practice parenting commitment
5. Parenting your anxious child	Common parent-child interaction patterns in anxiety-affected families Using ACT skills to change problematic patterns	Practice parenting commitment specific to autonomy granting and reducing anxious modeling
6. Self-care: there's only one you	Listening with compassion/Self-as-context (Defusing “failure” Creating a touchstone: Turning back to values/committed action	Keeping it going Making time for you

The intervention was specifically modified for parents of anxious children in two meaningful ways. First, content was dedicated to helping parents understand the impact of anxiety on parenting. Using experiential exercises and discussion, parents practiced accepting their own emotional reactions (e.g., anxiety, anger, frustration, exhaustion, etc.), defusing from catastrophic thoughts, and noticing unhelpful verbal rules (e.g., “Good parents do not allow their child to become distressed”) in response to their child's anxiety. Parents were also encouraged to reflect on the reactive behavioral solutions they had undertaken to alleviate their own discomfort when the child was anxious, which included rescuing the child, engaging in avoidance, accommodating or taking over all or part of the child's experience, chiding or punishing the child, or ignoring/invalidating or minimizing their anxious child's experience. In reflecting on these behavioral impulses, parents considered whether these behavioral impulses moved them towards or away from their own parenting values.

A second modification to the ACT-PAC protocol included content focused on supporting parents in using ACT to connect with their own parenting values when anxiety is present. Parents practiced making space for the discomfort that arose when their child was anxious, and making mindful behavioral choices. In doing so, parents thought deeply about how they could stay connected to their parenting values even when upset by their child's distress, which often looked like allowing their child to learn to tolerate or accept anxiety and still do things they love. This gave parents the opportunity to practice taking committed action towards their values instead of reactively attempting to alleviate their child's discomfort.

### 2.3 Measures—parent report

*Demographics Form*. Parents completed demographic information, including parent/child gender, race/ethnicity, and age.

#### 2.3.1 Parent functioning

*Depression, Anxiety, and Stress Scale* [DASS; (32)]. The DASS consists of 42 negative emotional symptoms of depression, anxiety, and stress. Parents rated the extent to which the symptom applied to them over the past week on a scale ranging from 0 (did not apply to me at all) to 3 (applied to me very much, or most of the time). Internal consistencies (coefficient alpha) for each scale for the DASS normative sample were: Depression 0.91; Anxiety 0.84; and Stress 0.90 (32).

*Quality of Life Enjoyment and Satisfaction Questionnaire-Short Form* [Q-LES-Q-SF; (33)]. The Q-LES-Q-SF is a 16-item rating scale which assesses satisfaction and functioning in the domains of social, leisure, household, emotional well-being, and physical in the past week. Parents rated items on a 5-point scale ranging from 1 (very poor) to 5 (very good). The internal consistency and test-retest coefficients of this questionnaire were 0.9 and 0.93, respectfully (34).

#### 2.3.2 ACT-related parent measures

*Multidimensional Experiential Avoidance Questionnaire* [MEAQ; (35)]. The MEAQ is a 62-item measure of experiential avoidance or the unwillingness to remain in contact with distressing feelings, thoughts, memories or other private experiences (22). The measure consists of 6 subscales: behavioral avoidance (e.g., “I go out of my way to avoid uncomfortable situations”), distress aversion (e.g., The key to a good life is never feeling any pain”), repression/denial (e.g., “I am able to ‘turn off’ my emotions when I don't want to feel”), distraction/suppression (e.g., When something upsetting comes up, I try very hard to stop thinking about it”), and distress endurance (e.g., “I am willing to suffer for the things that matter to me”). Parents rated items on a 6-point scale ranging from strongly disagree to strongly agree. The MEAQ subscales have demonstrated internal consistency in a validation sample of community adulst, with alphas averaging .87 (36).

*Cognitive Fusion Questionnaire* [CFQ; (37)]. The CFQ is a 7-item rating scale which assesses cognitive fusion or the tendency for behavior to be overly regulated and dominated by cognitive events. The measure is scored as a total score, with higher numbers reflecting greater fusion (e.g., “My thoughts cause me distress or emotional pain”). Gillanders et al. (37) reported a Cronbach's alpha of.90 from a student and community sample and.92 from a sample of people with work stress.

*ACT Parenting Measure:* This brief 4-item measure, designed specifically for this study measured parental report over the past week of (1) parental perceptions of match between the past week’s parenting behavior and their own parenting values, (2) the perceived impact of strong emotion on parenting, and (3) the perceived impact of negative thoughts on parenting.

Youth Functioning

*Child Behavior Checklist* [CBCL; (38)]. The CBCL is a 113-item standardized measure of children's emotional, behavioral, and social functioning. Items measured anxiety, social withdrawal, depression, obsession–compulsions, non-communicative behavior, hyperactivity, aggression and somatic complaints. Parents rated items on a scale ranging from 0 (note true) to 2 (very true or often true). Achenbach and Rescorla (38) report test-retest reliability (Pearson's r) of 0.8–0.94, and internal consistency (Cronbach's alpha) of 0.63–0.97.

*Screen for Child Anxiety Related Emotional Disorders* [SCARED; (39)]. The SCARED is a 41-item symptom inventory used to screen for anxiety disorders. Parents rated symptoms for children's panic disorder, generalized anxiety disorder, separation anxiety, social anxiety disorder, and school avoidance on a scale ranging from 0 (not true or hardly ever true) to 2 (very true or often true). Each subscale has shown internal consistency coefficient values ranging from .78 to .87 (39).

#### 2.3.3 Acceptability measures

*Client Satisfaction Questionnaire* [CSQ-8; (40)]. The Client Satisfaction Questionnaire is a reliable and valid 8-item self-report questionnaire that yields a measure of satisfaction with treatment. The scale ranges from 4 to 32, with higher scores indicating increased treatment satisfaction.

*Qualitative Feedback Interview.* The Qualitative Feedback Interview is a brief interview designed specifically for this study consisting of seven open-ended questions. Administered to the participating caregiver, this interview gathered participants’ (1) overall experience with the study, (2) perceived acceptability, strengths and weaknesses of the group treatment protocol, and (3) perceived acceptability and tolerability of the study assessments. The qualitative interview was designed to measure whether the participants found the intervention to be appropriate, fair, reasonable, and consistent with treatment expectations (41).

### 2.4 Measures—child report

#### 2.4.1 Youth functioning

*Child Behavior Checklist Youth Self Report* [CBCL-YSR; (42)]. The YSR is a self-report measure of behavioral and emotional problems in youth ages 11–18. It contains 113 items and eight sub-scales that measure symptoms of withdrawn, somatic complaints, anxiety and depression, social problems, thought problems, attention problems, aggressive behavior, and delinquent behaviors (42). Achenbach and Rescorla (38) report test-retest reliability (Pearson's r) of 0.67–0.91, internal consistency (Cronbach's alpha) of 0.71–0.95, and inter-rater reliability with the CBCL of 0.57–0.88.

*Screen for Child Anxiety Related Emotional Disorders* [SCARED; (39)]. Children also completed the 41-item child-report version of the SCARED. All subscales had internal consistency coefficients ranging from .78 to .87 (39).

## 3 Analyses

Study data were collected and managed using REDCap electronic data capture tools hosted at UMass Chan Medical School (43, 44). Double-data entry was used; all data entry discrepancies were resolved by consensus agreement. Quantitative data were analyzed as the change in value over the study period using a linear mixed model that accounts for the random effects associated with the treatment cohort (that is, the measures for participants in the same treatment cohort are likely correlated) and repeated measures within participants. For most measures, “study period” refers to pre-treatment and post-treatment. For the CFQ and ACT Parenting Measure, assessments were completed each week in addition to pre- and post-treatment. All analyses were done using the R Project for Statistical Computing (45), with contributed packages “lme4” (46) and “ggplot2” (47). Though uncorrected *p*-values are presented, a correction for False Discovery Rate (48) was applied to account for multiplicity in testing. Confidence intervals were estimated using the “Wald” method. Effect size was estimated from the test statistic of the fixed effect as $t/\sqrt{df_{error}}$.

## 4 Results

### 4.1 Preliminary analyses

One parent exceeded the clinical cut-off for depression (with a score of 12 or greater on the DASS). Five parents exceeded the clinical cut-off for anxiety (with a score of 5 or greater on the DASS).

### 4.2 Main analyses

**Figure 1:**
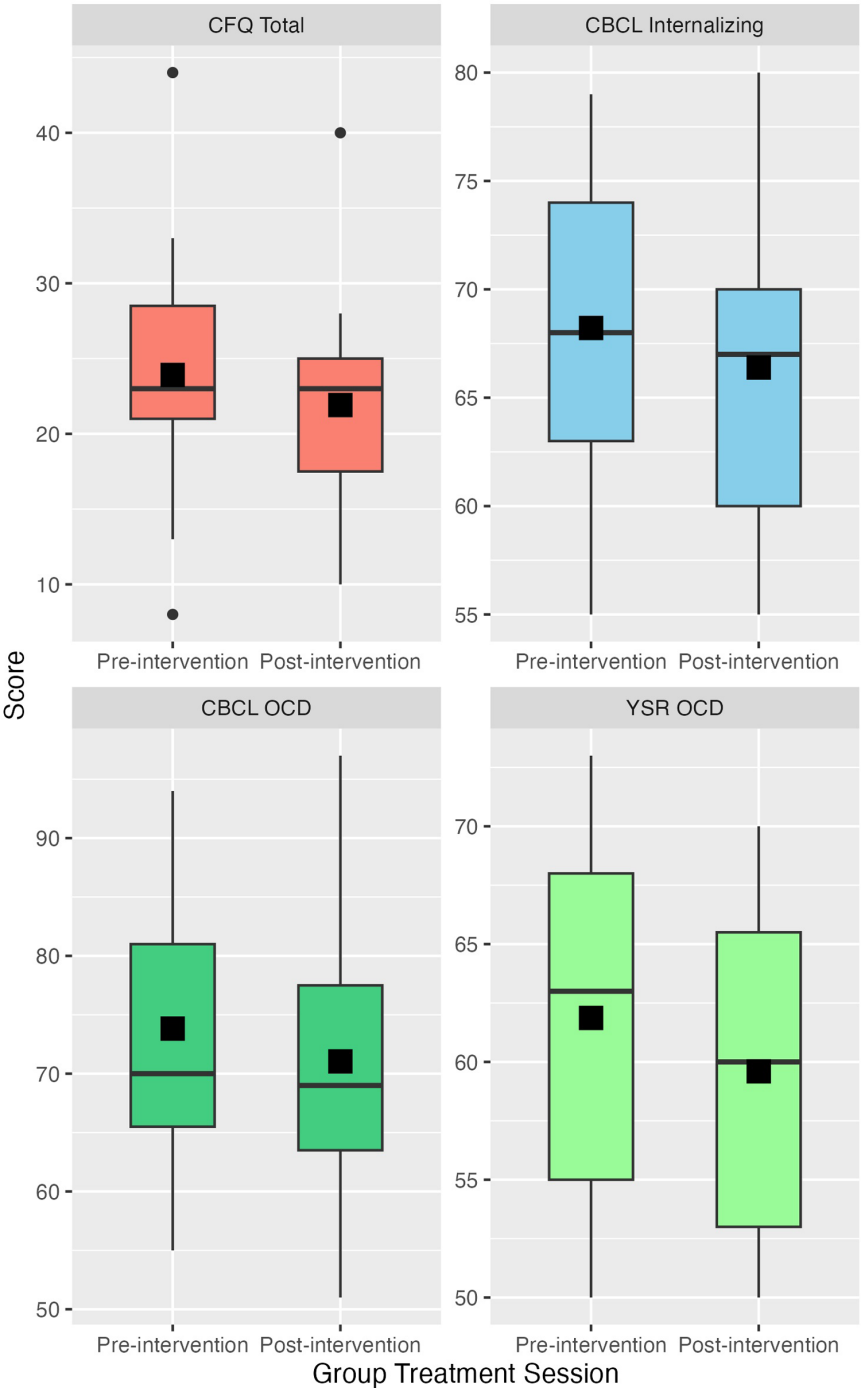
Change between pre-intervention and post-intervention for cognitive fusion, CBCL internalizing, CBCL obsessive compulsive disorder, and YSR obsessive compulsive disorder. Height of the box represents the interquartile range; median and mean are represented by the horizontal bar and filled square within each box, respectively.

**Figure 2:**
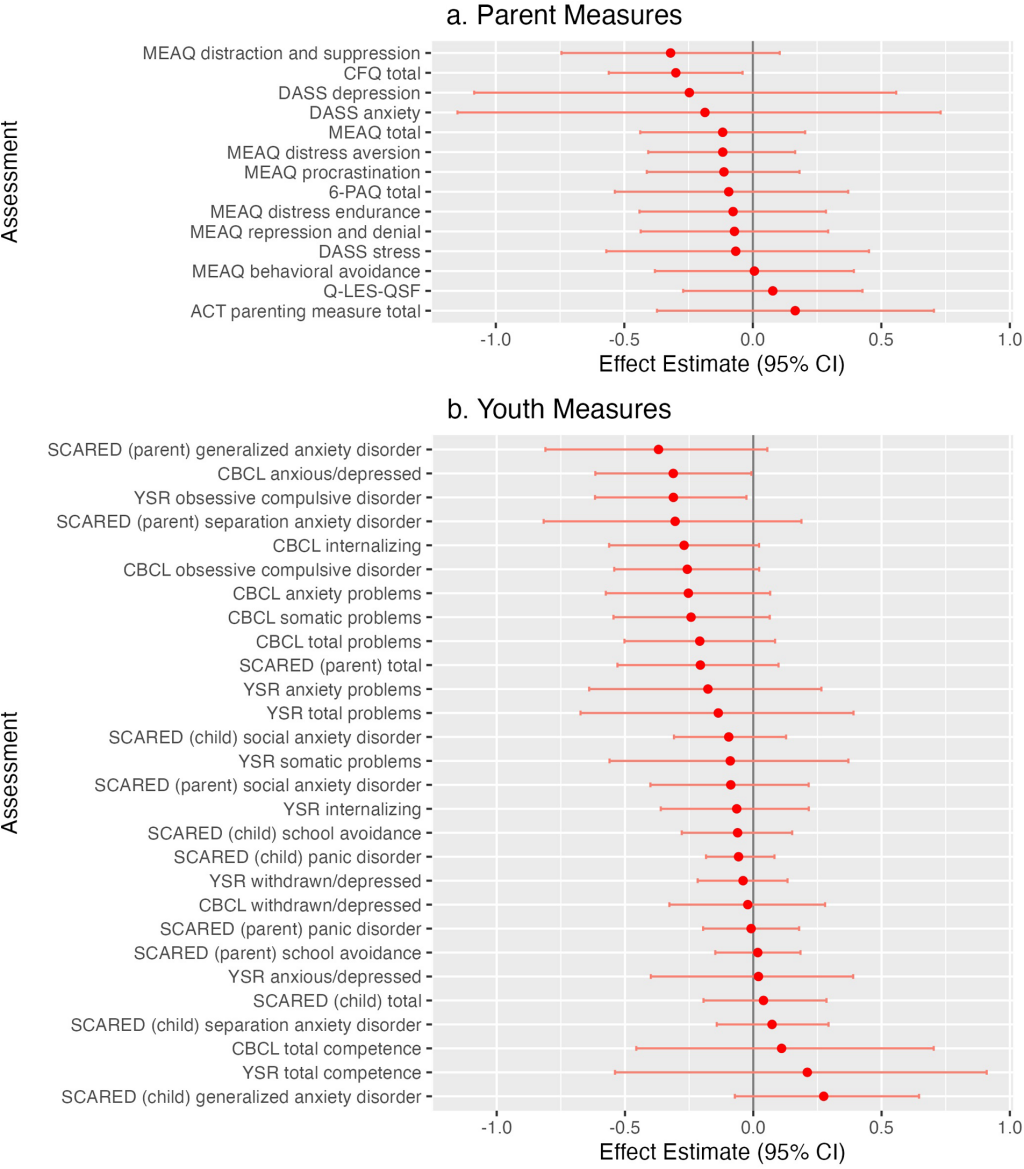
**(A)** Standardized effect estimates (child measures, sorted). **(B)** Standardized effect estimates (Parent Measures, sorted).

There were no significant changes from pre-treatment to post-treatment among the Parent Symptoms measures (see Table 2, Figure 1 and Figure 2A). The Cognitive Fusion Questionnaire (CFQ) Total score showed a 2 point reduction during the treatment period [95% CI: −3.7, −0.3; t(22) = −2.3, uncorrected *p* = 0.03]. The Distraction and Suppression scale of the MEAQ was 1.5 points lower post-treatement [95% CI: −3.5, 0.5; t(22) = −1.5, *p* = 0.15].

**Figure 3:**
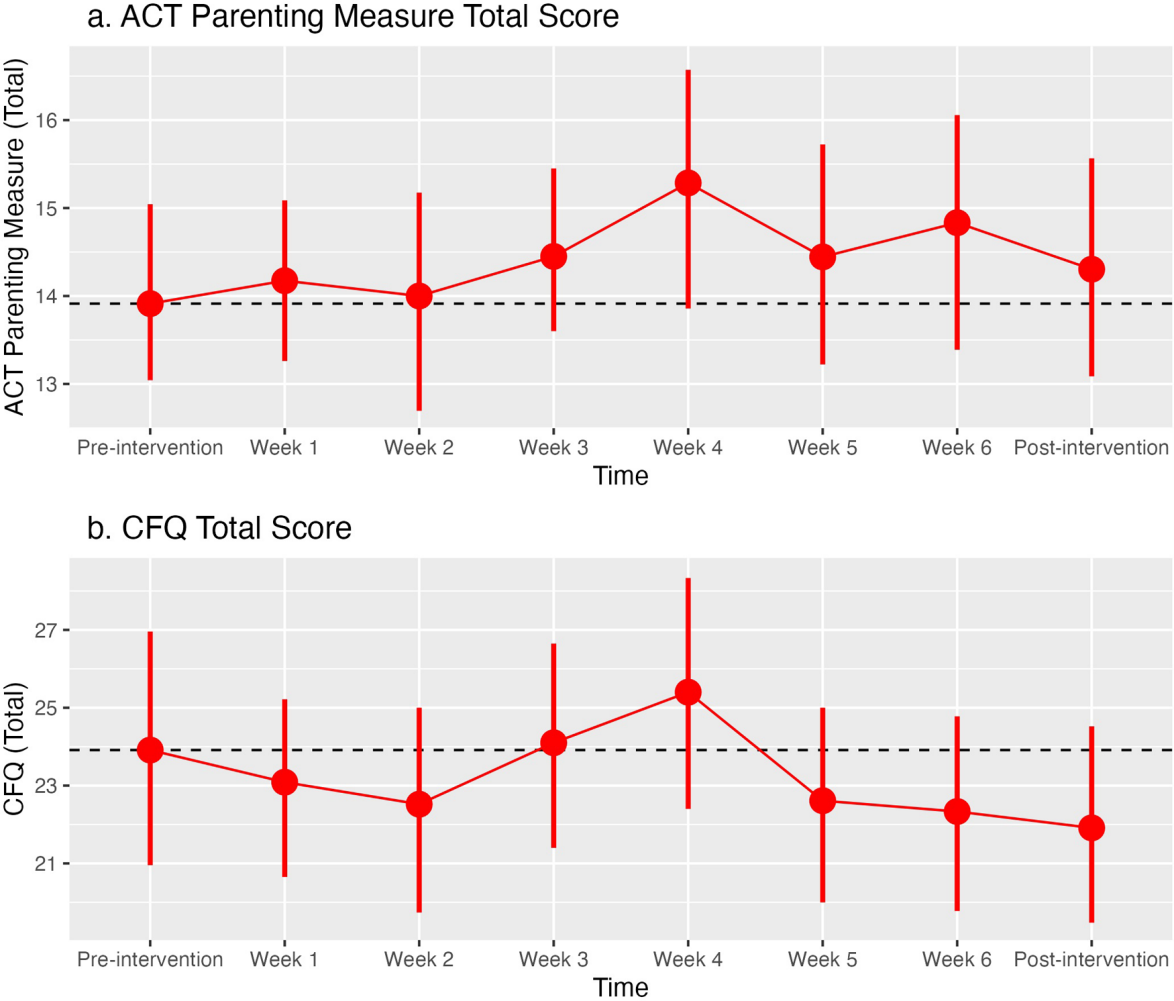
Average scores for measures obtained at each treatment session: **(A)** Acceptance and Commitment Therapy (ACT) Parenting Measure total score; **(B)** Cognitive Fusion Questionnaire (CFQ) total score. Error bars represent 95% confidence intervals.

Neither the ACT Parenting Measure nor the CFQ Total Score showed a significant change from pre-treatment across the weekly training sessions: the ACT Parenting measure increased 0.1 points at each assessment [95% CI: −0.8, 0.29; t(22.8) = 1.1, *p* = 0.3]; while the CFQ Total Score decreased 0.17 points at each assessment (95% CI: −0.4, 0.1); t(21.4) = −1.3, *p* = 0.2) ([see Figure 3, of scores at each week). However, when restricted to only the pre-treatment and post-treatment assessments, the CFQ Total score showed a 2 point reduction during the treatment period [95% CI: −3.7, −0.3; t(22) = −2.3, *p* = 0.03].

There were no significant changes from pre-treatment to post-treatment among the Child Symptoms measures (see Table 3 and Figure 2A). Modest effects (uncorrected *p*-values <0.1) were seen as the parent-rated CBCL t-scores for Internalizing symptoms were 1.8 points lower post-treatment [95% CI: −3.8, 0.1; t(22) = −1.8, *p* = 0.08] and the Obsessive Compulsive Disorder symptoms were 2.8 points lower post-treatment [95% CI: −5.7, 0.2; t(22) = −1.8, *p* = 0.08]. The child-rated YSR t-score for Obsessive Compulsive Disorder symptoms was also lower by 2.3 points [95% CI: −4.2, −0.4; t(3.1) = −2.3, *p* = 0.10].

**Table 3A:** Change between Pre-intervention and post-intervention for measures of parent functioning.

Parent symptoms measures	*n*	Pre-intervention		Post-intervention		Model estimates^a^				
		M	SD	M	SD	Estimate	(95% CI)	T	P	Effect size
DASS depression	23	4.4	4.7	3.7	4.6	−1	(−3.9, 1.9)	−0.7	0.55	−0.14
DASS anxiety	23	2.5	2.6	2.2	2.9	−0.3	(−2.0, 1.3)	−0.4	0.69	−0.09
DASS stress	23	10.7	6.2	10.3	7.5	−0.4	(−3.7, 2.9)	−0.2	0.83	−0.05
Q-LES-QSF	23	0.7	0.2	0.7	0.1	0	(−0.0, 0.1)	0.4	0.66	0.09
MEAQ behavioral avoidance	23	32.2	7.3	32.2	8.5	0	(−2.7, 2.8)	0	0.98	0.01
MEAQ distress aversion	23	42.3	8.8	41.2	10.8	−1.1	(−3.5, 1.3)	−0.9	0.44	−0.19
MEAQ procrastination	23	22.8	6.6	22	7.3	−0.7	(−2.6, 1.2)	−0.8	0.45	−0.16
MEAQ distraction and suppression	23	26.5	4.8	25	5.8	−1.5	(−3.5, 0.5)	−1.5	0.15	−0.31
MEAQ repression and denial	23	27.9	7.5	27.3	9.6	−0.6	(−3.4, 2.3)	−0.4	0.7	−0.08
MEAQ distress endurance	23	48	5.2	47.6	7	−0.4	(−2.4, 1.6)	−0.4	0.67	−0.09
MEAQ total	23	180.7	26.5	177.2	35.6	−3.4	(−12.6, 5.8)	−0.7	0.47	−0.15
CFQ total	23	23.9	7.6	21.9	6.4	−2	(−3.7, −0.3)	−2.3	0.03	−0.49
6-PAQ total	23	42.3	3.9	42	3.1	−0.3	(−1.6, 1.0)	−0.5	0.67	−0.1
ACT parenting measure total	23	13.9	2.6	14.3	3.1	0.4	(−0.9, 1.6)	0.6	0.55	0.13

^a^ Fixed effect estimates from model incorporating the random nested effect of treatment cohort.

**Table 3B:** Change between pre-intervention and post-intervention for measures of youth functioning.

Child symptoms measures	*n*	Pre-Intervention		Post-Intervention		Model Estimates†				
		M	SD	M	SD	Estimate	(95% CI)	T	P	Effect Size
CBCL total competence	22	44	9.9	44.6	8.9	1	(−3.5, 5.5)	0.4	0.71	0.09
CBCL internalizing	23	68.2	6.8	66.4	7.6	−1.8	(−3.8, 0.1)	−1.8	0.08	−0.38
CBCL anxious/depressed	23	69.5	8.1	67.2	8	−2.4	(−4.6, −0.1)	−2	0.12	−0.43
CBCL withdrawn/depressed	23	62.7	9.9	62.5	11.5	−0.2	(−3.2, 2.8)	−0.1	0.89	−0.03
CBCL somatic problems	23	63.8	10.4	61.6	8.5	−2.2	(−4.8, 0.4)	−1.7	0.11	−0.34
CBCL total problems	23	62.8	6.4	61.5	6.4	−1.3	(−3.0, 0.5)	−1.4	0.17	−0.3
CBCL anxiety problems	23	69.7	5.7	68.1	7.7	−1.6	(−3.6, 0.4)	−1.6	0.13	−0.33
CBCL obsessive compulsive disorder	23	73.8	11.5	71	11.3	−2.8	(−5.7, 0.2)	−1.8	0.08	−0.38
YSR total competence	15	46.9	9.4	48.5	7.8	1.5	(−3.2, 6.3)	0.6	0.56	0.16
YSR internalizing	15	19.4	11.2	18.9	11.4	−0.7	(−3.6, 2.1)	−0.5	0.66	−0.13
YSR anxious/depressed	15	62.3	10.2	62.6	8.9	0.2	(−3.0, 3.3)	0.1	0.92	0.03
YSR withdrawn/depressed	15	59.7	9.7	59.3	10.7	−0.4	(−2.1, 1.3)	−0.5	0.65	−0.12
YSR somatic problems	15	57.2	7.1	56.6	9.7	−0.7	(−4.1, 2.6)	−0.4	0.7	−0.11
YSR total problems	15	55.9	7.9	55	9.5	−1.1	(−5.1, 2.8)	−0.6	0.61	−0.15
YSR anxiety problems	15	64.3	8.3	63.1	7.8	−1.4	(−4.4, 1.7)	−0.9	0.45	−0.22
YSR obsessive compulsive disorder	15	61.9	7.8	59.6	7.2	−2.3	(−4.2, −0.4)	−2.3	0.1	−0.61
SCARED (child) total	23	28.4	17.6	28.9	17.9	0.7	(−3.0, 4.4)	0.4	0.74	0.08
SCARED (child) panic disorder	23	6.4	6.7	6	7	−0.4	(−1.2, 0.4)	−1	0.41	−0.2
SCARED (child) generalized anxiety disorder	23	8	4.9	9.2	4.8	1.3	(−0.2, 2.8)	1.7	0.19	0.35
SCARED (child) separation anxiety disorder	23	3.7	3.6	3.9	3.7	0.3	(−0.5, 1.0)	0.7	0.5	0.14
SCARED (child) social anxiety disorder	23	7.9	5.3	7.4	5	−0.5	(−1.5, 0.5)	−0.9	0.37	−0.19
SCARED (child) school avoidance	23	2.5	1.9	2.4	2.3	−0.1	(−0.6, 0.3)	−0.5	0.63	−0.11
SCARED (parent) total	23	29.6	15.3	26.6	16.4	−3.1	(−7.4, 1.1)	−1.4	0.25	−0.3
SCARED (parent) panic disorder	23	5.2	4.9	5.2	5.6	0	(−1.0, 0.9)	−0.1	0.93	−0.02
SCARED (parent) generalized anxiety disorder	23	10	5.1	8.4	4.5	−1.7	(−3.5, 0.1)	−1.9	0.16	−0.39
SCARED (parent) separation anxiety disorder	23	4.2	4.4	3.2	3.3	−1.1	(−2.7, 0.5)	−1.3	0.28	−0.28
SCARED (parent) social anxiety disorder	23	7.7	4.9	7.3	5.2	−0.4	(−1.8, 0.9)	−0.6	0.58	−0.13
SCARED (parent) school avoidance	23	2.5	2.5	2.5	2.6	0	(−0.4, 0.4)	0.2	0.83	0.04

†Fixed effect estimates from model incorporating the random nested effect of treatment cohort.

Parent-rated scores on the SCARED were generally lower post-treatment than the child-rated scores. Though there were no significant differences between pre-treatment and post-treatment ratings, the Generalized Anxiety Disorder score was reduced on the parent-rated SCARED [−1.7 points, 95% CI: −3.5, 0.1; t(2.9) = −1.9, *p* = 0.16], but increased on the Child-rated SCARED [1.3 points, 95% CI: −0.2, 2.8; t(2.9) = 1.7, *p* = 0.19]. A similar pattern was seen for the Anxious/Depressed scale between the parent-rated CBCL and the child-rated YSR. The T scores were −2.4 points between pre-treatment and post-treatment for the parent ratings [95% CI: −4.6, −0.1; t(3.4) = −2.0, *p* = 0.12] and 0.2 points for the child-rated YSR [95% CI: −3.0, 3.3; t(2.5) = 0.1, *p* = 0.9].

### 4.3 Acceptability and feasibility

**Figure 4:**
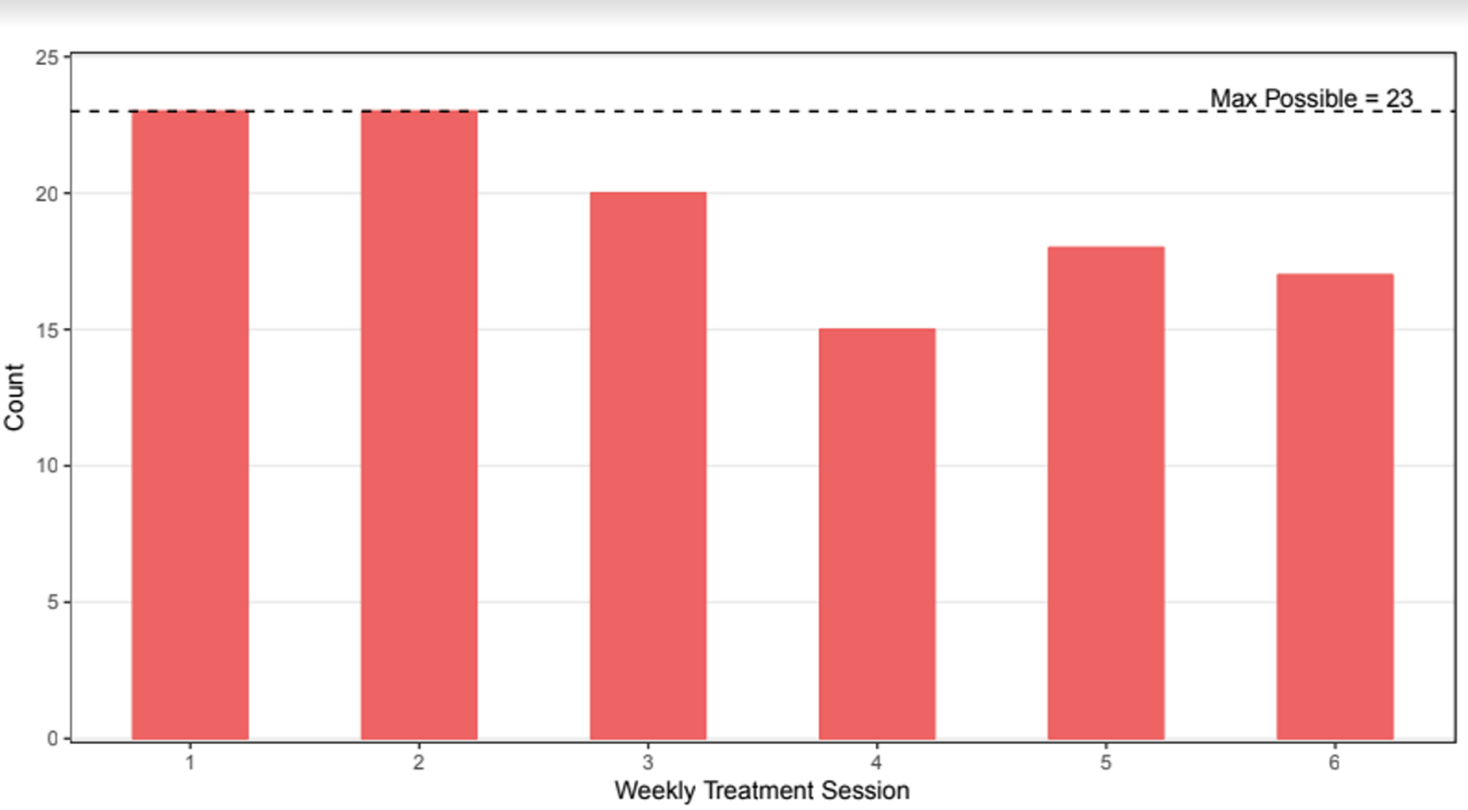
Parent attendance at weekly sessions.

Results indicated that parents were satisfied with the experience of ACT-PAC as reported on the Client Satisfaction Questionnaire [CSQ-8 (40)], *M* = 25.39, *SD* = 4.76 (scores range from 16 to 32). The group intervention was shown to be feasible for parents to attend. Parents attended 5 of 6 weekly sessions on average, and 91% of parents attended 4 or more sessions (see Figure 4).

## 5 Discussion

Overall, the results suggest that the ACT-PAC group may be acceptable and feasible and thus appropriate for large-scale evaluation, implementation, and dissemination. Results indicated that the intervention may successfully decrease parent's cognitive fusion, allowing them to approach their thoughts about their child's anxiety disorder with greater psychological flexibility. Such findings replicate recently published pilot work showing that cognitive fusion decreased following ten group sessions of Acceptance and Commitment Therapy as parent counseling for parents of children with a variety of psychiatric problems (49). These findings are also consistent with work by Blackledge and Hayes (30) showing that a 2-day group ACT workshop with parents of autistic children produced reduced cognitive fusion that later played a role in decreasing parental depression. There are important clinical implications to this work. When parents are less fused to their own thoughts about their child's anxiety, they may be more supportive during their child's treatment by encouraging exposure to anxiety-provoking situations rather than avoidance, or providing less reassurance when their child is struggling. Results also suggested, although these findings were marginal, that the intervention may successfully reduce children's internalizing (anxiety and depression) and obsessive-compulsive disorder symptoms, at least as perceived by parents, by virtue of parents learning to think about and respond to their child's anxiety in new ways. This work extends a growing body literature that has found that an ACT intervention designed for parents produces favorable outcomes in children [e.g., (21, 29)]. Interestingly, unlike other ACT interventions for parents [e.g., (30)] the intervention did not produce a reduction in experiential avoidance. Unlike for cognitive fusion, it may be particularly difficult for parents to remain in contact with distressing feelings, thoughts, memories or other private experiences in the absence of between-session support for moments in which they are acutely stressed, and may be most likely to fall back on patterns of avoidance. In addition, there were no changes in parental quality of life or clinical outcomes, though it is worth noting that most of the participants in the study were not highly distressed according to our measures of depression, stress and anxiety. Although some research on other parent populations [e.g., parents of children diagnosed with autism; (30)] has shown that Acceptance and Commitment Therapy can improve psychological outcomes, others have found improvements in ACT processes (e.g., mindfulness, acceptance, valued living and cognitive defusion) but not parent stress, anxiety and depression (50). Such findings may suggest that parents’ ability to work with symptoms improve over treatment, and that even if symptoms are present, they may be less disruptive. This is clinically relevant as parents caring for a child with anxiety may themselves experience stress, anxiety or depression and so equipping parents with the skills to live with these experiences may be more significant than eradicating the symptoms altogether.

### 5.1 Limitations and future directions

There were several noteworthy limitations to this study. First, is the homogenous sample, which was not representative of the general population of parents of anxious children, in that it was white and highly educated. Further, our recruitment through a Pediatric OCD and Anxiety Disorders Clinic raises the possibility that patients in the sample had high treatment access, further limiting the generalizability of our study findings. It remains to be studied whether ACT-PAC would be acceptable to parents of different cultural groups or whether the effects of the group may differ among a diverse set of families, in terms of race/ethnicity, socioeconomic status, and presenting problems. Replication with other samples is a necessary next step.

Second, although client satisfaction surveys and high attendance rates point to an intervention that is both feasible to implement and acceptable to clients, future research, that may take the form of a focus group with participating families, should obtain additional parent feedback on the intervention and on study protocols. In particular, given that the intervention was delivered as a weekly group, without between-session support to help parents implement recommended changes in moments when they are acutely stressed, it would be important to assess whether participants had trouble remembering to use the techniques during times of acute distress. Newly developed technology (e.g., wearable biosensors) can alert parents when their physiological stress levels are rising and provide immediate reminders (via smartphone) to use the newly learned techniques in these moments of stress when the techniques might be most needed. Future research may explore with parents how this mobile technology might further support the usefulness of ACT-PAC.

Third, for the vast majority of our measures, our data only had two timepoints (pre/post). As a result, we were limited to only providing information about change, and could not assess additional information about the shape of change (e.g., linear, non-linear) or the timing of change. Further, in the absence of a comparison group (e.g., waitlist control or psychoeducational control group), we cannot draw any conclusions about the “active ingredient” driving intervention effects. It is entirely possible that the preliminary effects demonstrated in this study were due to the social support received from the interventions’ group setting or merely just the passage of time. A more rigorous study design in the form of a randomized clinical trial is necessary to better understand the mechanism of change to to explicitly determine whether the effects are due to non-specific factors (e.g., group support, opportunities for self-disclosure) or specific ACT content (e.g., mindfulness, cognitive defusion). Fourth, this study does not provide information about change in targeted parenting behaviors (e.g., autonomy support) through treatment. While our study included both child clinical outcomes and ACT-related measures, we did not measure whether the intervention changed parenting behaviors. Future research should systematically evaluate change in parent behavior during parent-child interactions as a result of this intervention and address the mechanisms through which ACT-PAC may be exerting its influence [e.g., by enhancing psychological flexibility and acceptance (22);]. Future research should also consider including multi-informant clinical outcomes, especially in light of the current findings showing increases in child-reported generalized anxiety disorder symptoms but decreases in the same symptoms when parents were the informant (although neither increase nor decrease reached statistical significance). This intervention may produce particularly salient effects for parent reported clinical outcomes, as the intervention may impact the lens through which parents are viewing their child.

In summary, in light of research highlighting that up to 40% of youth don't meaningfully benefit from treatment (8), this study suggests that ACT-PAC may uniquely address parent-child processes that may be impeding treatment progress. Results suggest that ACT may benefit anxiety affected families, improving both parents and children, and help families effectively engage in valued pursuits.

## Data Availability

The datasets presented in this article are not readily available because HIPPA. Requests to access the datasets should be directed to jrafteryhelmer@worcester.edu.

## Footnotes

^1^Coyne LW. *ACT for Parents*. Department of Psychology, Suffolk University (2014).

## References

[1] Costello EJ Mustillo S Erkanli A Keeler G Angold A. Prevalence and development of psychiatric disorders in childhood and adolescence. Arch Gen Psychiatry. (2003) 4:469–86. 10.1001/archpsyc.60.8.837 12912767

[2] Kessler RC Petukhova M Sampson NA Zaslavsky AM Wittchen HU. Twelve-month and lifetime prevalence and lifetime morbid risk of anxiety and mood disorders in the United States. Int J Methods Psychiatr Res. (2012) 21(3):169–84. 10.1002/mpr.1359 22865617 PMC4005415

[3] Avenevoli S Stolar M Li J Dierker L Ries MK. Comorbidity of depression in children and adolescents: models and evidence from a prospective high-risk family study. Biol Psychiatry. (2001) 38:129–37. 10.1016/S0006-3223(01)01142-8 11430849

[4] Hirshfeld-Becker DR Micco JA Simoes NA Henin A. High risk studies and developmental antecedents of anxiety disorders. Am J Med Genet C Semin Med Genet. (2008) 148(2):99–117. 10.1002/ajmg.c.30170 18409200

[5] Kushner MG Sher KJ Beitman BD. The relation between alcohol problems and the anxiety disorders. Am J Psychiatry. (1990) 147:685–95. 10.1176/ajp.147.6.685 2188513

[6] Kushner MG Wall MM Krueger RF Sher KJ Maurer E Thuras P Alcohol dependence is related to overall internalizing psychopathology load rather than to particular internalizing disorders: evidence from a national sample. Alcohol Clin Exp Res. (2012) 36:325–31. 10.1111/j.1530-0277.2011.01604.x 21895708 PMC3235250

[7] Schleider JL Vélez CE Krause ED Gillham J. Perceived psychological control and anxiety in early adolescents: the mediating role of attributional style. Cognit Ther Res. (2014) 38(1):71–81. 10.1007/s10608-013-9573-9

[8] Kendall PC Settipani CA Cummings CM. No need to worry: the promising future of child anxiety research. J Clin Child Adolesc Psychol. (2012) 41:103–15. 10.1080/15374416.2012.632352 22233250

[9] Barber BK. Parental psychological control: revisiting a neglected construct. Child Dev. (1996) 67(6):3296–319. 10.1111/j.1467-8624.1996.tb01915.x 9071782

[10] Steinberg L Elmer JD Mounts NS. Authoritative parenting, psychosocial maturity, and academic success among adolescents. Child Dev. (1989) 60:1424–36. 10.2307/1130932 2612251

[11] Moore PS Whaley SE Sigman M. Interactions between mothers and children: impacts of maternal and child anxiety. J Abnorm Psychol. (2004) 113:471–6. 10.1037/0021-843X.113.3.471 15311992

[12] Manassis K Lee TC Bennett K Zhao XY Mendlowitz S Duda S Types of parental involvement in CBT with anxious youth: a preliminary meta-analysis. J Consult Clin Psychol. (2014) 82(6):1163. 10.1037/a0036969 24841867

[13] Murrell AR Wilson KG LaBorde CT Drake CE Rogers LJ. Relational responding in parents. Behav Anal Today. (2008) 9:196–214. 10.1037/h0100659

[14] Mendlowitz SL Manassis K Bradley S Scapillato D Miezitis S Shaw BE. Cognitive-behavioral group treatments in childhood anxiety disorders: the role of parental involvement. J Am Acad Child Adolesc Psychiatry. (1999) 38:1223–9. 10.1097/00004583-199910000-00010 10517054

[15] Spence SH Donovan C Brechman-Toussaint M. The treatment of childhood social phobia: the effectiveness of social skills training-based cognitive-behavioral intervention, with and without parental involvement. J Child Psychol Psychiatry. (2000) 41:713–26. 10.1111/1469-7610.00659 11039684

[16] Lebowitz ER Omer H Hermes H Scahill L. Parent training for childhood anxiety disorders: the SPACE program. Cogn Behav Pract. (2014) 21(4):456–69. 10.1016/j.cbpra.2013.10.004

[17] Freeman JB Garcia AM Coyne L Ale C Przeworski A Himle M Early childhood OCD: preliminary findings from a family-based cognitive-behavioral approach. J Am Acad Child Adolesc Psychiatry. (2008) 47(5):593–602. 10.1097/CHI.0b013e31816765f9 18356758 PMC2820297

[18] Freeman J Sapyta J Garcia A Compton S Khanna M Flessner C Family-based treatment of early childhood OCD: the pediatric OCD treatment study junior (POTS jr.) randomized controlled trial. JAMA Psychiatry. (2014) 71(6):689–98. 10.1001/jamapsychiatry.2014.170 24759852 PMC4511269

[19] Lebowitz E Marin C Martino A Shimshoni Y Silverman W. Parent-based treatment as efficacious as cognitive-behavioral therapy for childhood anxiety: a randomized noninferiority study of supportive parenting for anxious childhood emotions. J Am Acad Child Adolesc Psychiatry. (2019) 59:362–72. 10.1016/j.jaac.2019.02.014 30851397 PMC6732048

[20] Cheron DM Ehrenreich JT Pincus DB. Assessment of parental experiential avoidance in a clinical sample of children with anxiety disorders. Child Psychiatry Hum Dev. (2009) 40:383–403. 10.1007/s10578-009-0135-z 19280337

[21] Coyne LW Wilson KG. The role of cognitive fusion in impaired parenting: an RFT analysis. Int J Psychol Psychol Ther. (2004) 4:469–86.

[22] Hayes SC Strosahl KD Wilson KG. Acceptance and Commitment Therapy: An Experiential Approach to Behavior Change. New York, NY: Guilford Press (1999).

[23] Raftery-Helmer JN Moore PS Coyne L Reed KP. Changing problematic parent–child interaction in child anxiety disorders: the promise of acceptance and commitment therapy (ACT). J Contextual Behav Sci. (2016) 5(1):64–9. 10.1016/j.jcbs.2015.08.002

[24] Arch JJ Ayers CR. Which treatment worked better for whom? Moderators of group cognitive behavioral therapy versus adapted mindfulness based stress reduction for anxiety disorders. Behav Res Ther. (2013) 51(8):434–42. 10.1016/j.brat.2013.04.004 23747582

[25] Forman EM Herbert JD Moitra E Yeomans PD Geller PA. A randomized controlled effectiveness trial of acceptance and commitment therapy and cognitive therapy for anxiety and depression. Behav Modif. (2007) 31(6):772–99. 10.1177/0145445507302202 17932235

[26] Wetherell JL Afari N Rutledge T Sorrell JT Stoddard JA Petkus AJ. A randomized, controlled trial of acceptance and commitment therapy and cognitive-behavioral therapy for chronic pain. Pain. (2011) 152(9):2098–107. 10.1016/j.pain.2011.05.016 21683527

[27] Swain J Hancock K Dixon A Koo S Bowman J. Acceptance and commitment therapy for anxious children and adolescents: study protocol for a randomized controlled trial. Trials. (2013) 14:140–52. 10.1186/1745-6215-14-140 23672442 PMC3662565

[28] Hancock KM Swain J Hainsworth CJ Dixon AL Koo S Munro K. Acceptance and commitment therapy versus cognitive behavior therapy for children with anxiety: outcomes of a randomized controlled trial. J Clin Child Adolesc Psychol. (2018) 47(2):296–31. 10.1080/15374416.2015.1110822 26998803

[29] Whittingham K Sanders M McKinlay L Boyd R. Parenting intervention combined with acceptance and commitment therapy. Process of change. J Child Fam Stud. (2019) 28:1673–80. 10.1093/jpepsy/jsv118 26702629 PMC4888113

[30] Blackledge JT Hayes SC. Using acceptance and commitment training in the support of parents of children diagnosed with autism. Child Fam Behav Ther. (2006) 28:1–18. 10.1300/J019v28n01_01

[31] Corti C Pergolizza F Vanzin L Cargasacchi G Villa L Pozzi M Acceptance and commitment therapy-oriented parent-training for parents of children with autism. J Child Fam Stud. (2018) 27:2887–900. 10.1007/s10826-018-1123-3

[32] Lovibond PF Lovibond SH. The structure of negative emotional states: comparison of the depression anxiety stress scales (DASS) with the beck depression and anxiety inventories. Behav Res Ther. (1995) 33(3):335–43. 10.1016/0005-7967(94)00075-U 7726811

[33] Endicott J Nee J Harrison W Blumenthal R. Quality of life enjoyment and satisfaction questionnaire: a new measure. Psychopharmacol Bull. (1993) 29(2):321–6. 8290681

[34] Stevanovic D. Quality of life enjoyment and satisfaction questionnaire-short form for quality of life assessments in clinical practice: a psychometric study. J Psychiatr Ment Health Nurs. (2011) 18(8):744–50. 10.1111/j.1365-2850.2011.01735.x 21896118

[35] Gámez W Chmielewski M Kotov R Ruggero C Suzuki N Watson D. The brief experiential avoidance questionnaire: development and initial validation. Psychol Assess. (2014) 26(1):35–45. 10.1037/a0034473 24059474

[36] Gámez W Chmielewski M Kotov R Ruggero C Watson D. Development of a measure of experiential avoidance: the multidimensional experiential avoidance questionnaire. Psychol Assess. (2011) 23(3):692–713. 10.1037/a0023242 21534697

[37] Gillanders DT Bolderston H Bond FW Dempster M Flaxman PE Campbell L The development and initial validation of the cognitive fusion questionnaire. Behav Ther. (2014) 45(1):83–101. 10.1016/j.beth.2013.09.001 24411117

[38] Achenbach TM Rescorla L. Manual for the ASEBA School-age Forms & Profiles: An Integrated System of Multi-Informant Assessment. Burlington, VT: Saeb (2001).

[39] Birmaher B Brent DA Chiappetta L Bridge J Monga S Baugher M. Psychometric properties of the screen for child anxiety related emotional disorders (SCARED): a replication study. J Am Acad Child Adolesc Psychiatry. (1999) 38(10):1230–6. 10.1097/00004583-199910000-00011 10517055

[40] McMurtry SL Hudson WW. The client satisfaction inventory: results of an initial validation study. Res Soc Work Pract. (2000) 10(5):644–63. 10.1177/104973150001000506

[41] Kazdin AE. Acceptability of time out from reinforcement procedures for disruptive child behavior. Behav Ther. (1980) 11:329–44. 10.1016/S0005-7894(80)80050-5

[42] Achenbach TM. The Child Behavior Checklist and related instruments. In Maruish ME, editor. The Use of Psychological Testing for Treatment Planning and Outcome Assessment. 2nd ed. Mahwah, NJ: Lawrence Erlbaum Associates Publishers (1999). p. 429–66.

[43] Harris PA Taylor R Thielke R Payne J Gonzalez N Conde JG. Research electronic data capture (REDCap)–a metadata-driven methodology and workflow process for providing translational research informatics support. J Biomed Inform. (2009) 42(2):377–81. 10.1016/j.jbi.2008.08.010 18929686 PMC2700030

[44] Harris PA Taylor R Minor BL Elliott V Fernandez M O'Neal L The REDCap consortium: building an international community of software platform partners. J Biomed Inform. (2019) 95:103208. 10.1016/j.jbi.2019.103208 31078660 PMC7254481

[45] R Core Team. R: A Language and Environment for Statistical Computing. Vienna, Austria: R Foundation for Statistical Computing (2023). Available online at: https://www.R-project.org

[46] Bates D Maechler M Bolker B Walker S. Fitting linear mixed-effects models using lme4. J Stat Softw. (2015) 67(1):1–48. 10.18637/jss.v067.i01

[47] Wickham H. ggplot2: Elegant Graphics for Data Analysis. New York: Springer (2016). Available online at: https://ggplot2.tidyverse.org

[48] Benjamini Y Hochberg Y. Controlling the false discovery rate: a practical and powerful approach to multiple testing. J R Stat Soc Ser B. (1995) 57:289–300. 10.1111/j.2517-6161.1995.tb02031.x11682119

[49] Bodden DHM Matthijssen D. A pilot study examining the effect of acceptance and commitment therapy as parent counseling. J Child Fam Stud. (2021) 30(4):978–88. 10.1007/s10826-021-01926-2

[50] Ahola Kohut S Martincevic I Turrell SL Church PC Walters TD Weiser N Online acceptance and commitment therapy and nutrition workshop for parents of children with inflammatory bowel disease: feasibility, acceptability, and initial effectiveness. Children. (2021) 8(5):396. 10.3390/children8050396 34069305 PMC8156170

